# Clinical Validation of the Autism Behavior Inventory: Caregiver-Rated Assessment of Core and Associated Symptoms of Autism Spectrum Disorder

**DOI:** 10.1007/s10803-019-03965-7

**Published:** 2019-03-19

**Authors:** Abigail Bangerter, Seth Ness, David Lewin, Michael G. Aman, Anna J. Esbensen, Matthew S. Goodwin, Geraldine Dawson, Robert Hendren, Bennett Leventhal, Fred Shic, Mark Opler, Kai Fai Ho, Gahan Pandina

**Affiliations:** 1 Department of Neuroscience, Janssen Research & Development, LLC, Titusville, NJ USA; 2 Department of Psychology, Ohio State University, 175C McCampbell, 1581 Dodd Drive, Columbus, OH USA; 3 Division of Developmental and Behavioral Pediatrics, Cincinnati Children’s Hospital Medical Center, 3430 Burnet Avenue, ML 4002, Cincinnati, OH USA; 4 Department of Health Sciences, Bouvé College of Health Sciences, Northeastern University, 312E Robinson Hall, 360 Huntington Avenue, Boston, MA USA; 5 Duke Center for Autism and Brain Development, Duke University, 2608 Erwin Road, Suite 300, Durham, NC USA; 6 Department of Psychiatry and the Weill Institute for Neuroscience, University of California, San Francisco, 401 Parnassus Ave, San Francisco, CA USA; 7 Present Address: Center for Child Health, Behavior and Development, Seattle Children’s Research Institute, Seattle, WA USA; 8 Present Address: Department of Pediatrics, University of Washington, Seattle, WA USA; 9 MedAvante-ProPhase, Inc, NYU School of Medicine, 3 Park Avenue Floors 28, 37, New York, NY USA; 10 STAT-TU Inc, Toronto, Canada; 11 Benioff Children’s Hospital, University of California, San Francisco,, San Francisco, CA USA; 12 Yale Child Study Center, Hartford, CT USA; 13 Present Address: Statistically Speaking Consulting, LLC, Chicago, IL USA

**Keywords:** Autism spectrum disorder, Rating scales and instruments, Assessment, Clinical trials, Caregiver-reported outcomes

## Abstract

There is a need for measures to track symptom change in autism spectrum disorder (ASD). We conducted a validation study on a revised version of the Autism Behavior Inventory (ABI), and a short form (ABI-S). Caregivers of individuals (6–54 years) with confirmed diagnoses of ASD (N = 144) completed the ABI and other rating scales at 4 time points. Scale consistency for each domain, 3–5 day test–retest reliability, and construct validity, determined by comparison to pre-specified scales, were all good. Change in the ABI was congruent with changes in other instruments. Collectively, results suggest incipient suitability of the ABI as a measure of changes in core and associated symptoms of ASD.

*Trial Registration* NCT02299700.

**Electronic supplementary material:**

The online version of this article (10.1007/s10803-019-03965-7) contains supplementary material, which is available to authorized users.

Measurement of core and associated features of ASD is complicated by developmental and phenotypic heterogeneity. There is an absence of reliable, sensitive endpoints for measuring clinically relevant changes in core symptoms of the disorder (Anagnostou et al. 2015; Aman et al. 2015; McConachie et al. 2015), which limits the development and evaluation of novel treatments that target core symptoms of ASD.

In recent reviews of rating scales for use as clinical endpoints, scales were classified on their clinical relevance and psychometric properties (Anagnostou et al. 2015; Lecavalier et al. 2014; Scahill et al. 2015). Examples of measures deemed to show high relevance for ASD and reasonably strong psychometric properties included: Child and Adolescent Symptom Inventory Anxiety Domain (CASI-Anxiety) (Hallett et al. 2013; Sukhodolsky et al. 2008), Repetitive Behavior Scale—Revised (RBS-R) (Bodfish et al. 1999), and the Aberrant Behavior Checklist (ABC) (Aman et al. 2004; Aman and Singh 2017). However, these scales tend to focus on specific features of ASD or were not designed to capture core features of ASD with granularity. Therefore, two or more assessment approaches might be needed to measure all relevant concepts in ASD. Additionally, not all scales are suitable for use in children and adults. For example, the Autism Impact Measure (Kanne et al. 2014; Mazurek et al. 2018) has been recently developed to assess core autism symptoms in children with ASD.

There remains a need for efficient scales able to document short-term sensitivity to change in core and associated symptoms of ASD, and which are validated in well characterized samples of individuals with ASD across a range of ages, representative of participants who might be involved in clinical trials.

To address this gap, the Autism Behavior Inventory (ABI) was recently developed as a novel, web-based, parent or caregiver rating scale for assessing ASD core symptoms and associated behaviors over a 1-week recall period (Fig. 1). Our aim was to create a psychometrically sound and sensitive outcome measure for ASD clinical trials and other interventional studies. The scale is aimed to be suitable for caregivers of people with ASD, age 3 through adulthood. The design, development, and initial psychometric properties of the ABI v 1.0, and the short form (ABI-S) are described elsewhere (Bangerter et al. 2017).

**Fig. 1:**
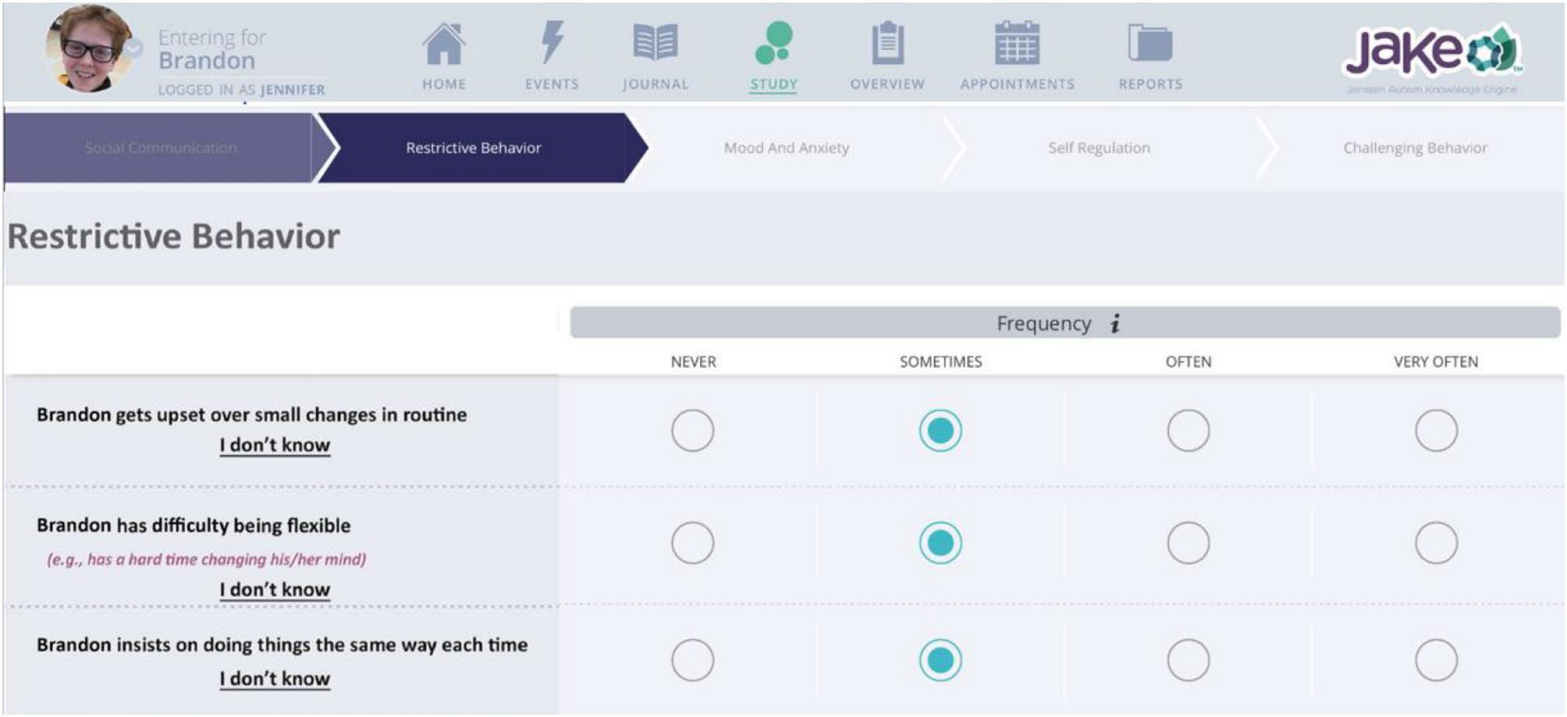
Sample ABI

In brief, the ABI was developed through an iterative process involving public-health and clinician experts, statistical validation, and parent feedback (Fig. 2). The clinical experts provided input to conceptualize the ABI by generating items, refining item wording, and evaluating completeness of item coverage across ASD domains; they also performed initial assessments of clarity and readability. After selection, the items were assigned to groups, forming domains and sub-domains of the ABI, which were confirmed with factor analysis in a sample (n = 353) of online survey responses. Reliability and validity of the 93-item ABI in a small clinical sample (n = 30) resulted in a reduction of items to a 73-item scale (Bangerter et al. 2017) and a 35 item short form. Short form items were selected based on their statistical performance, and clinical expert feedback through a Delphi process which required consideration of items that were likely to be of significance to caregivers, and most likely to show signs of change in the short term. Following consultation with the Food and Drug Administration (FDA), and a series of cognitive interviews, aimed at assessing caregiver understanding and perceived relevance of items, the scales were further refined and reduced (Pandina et al. 2018). The ABI v 1.1 is a 62-item scale, and the ABI-S v 1.1 has 24 items.

**Fig. 2:**
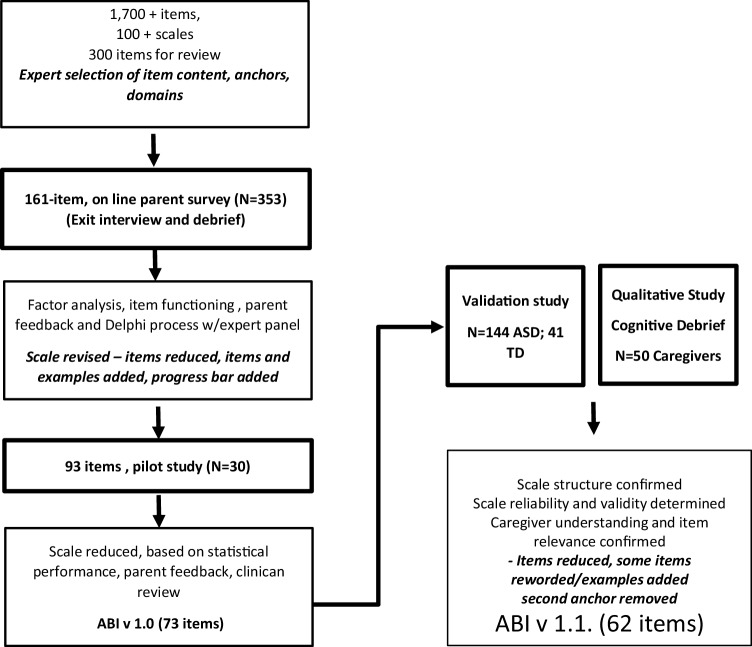
Development of the Autism Behaviour Inventory (ABI) Scale

This current observational study was designed to evaluate psychometric properties of the ABI (Table 1) and ABI-S in a larger, independent cohort of individuals with ASD (n = 144).

**Table 1:** Autism Behavior Inventory

ABI domain/item	Anchor type	ASD baseline Mean (SD) N = 140	TD Mean (SD) N = 34
Core ASD symptoms			
Social communication			
1. Shows appropriate affection towards familiar people	Q	0.8 (0.89)	0.0 (0.17)
2. **Shows an interest in what other people are doing**	Q	1.0 (0.90)	0.0 (0.17)
3. **Responds to attempts to initiate social interaction**	Q	1.2 (0.83)	0.0 (0.0)
4. Gives things to others in order to get help	Q	0.9 (0.99)	0.0 (0.17)
5/6. Engages in make believe play with another person*	Q	1.4 (1.24)	0.7 (1.31)
7. **Is able to take turns in conversation**	Q	1.2 (0.83)	0.1 (0.24)
8. Directs facial expression towards other people to communicate feelings	Q	1.2(1.07)	0.1 (0.41)
9. Offers information about his/her own thoughts or feelings	Q	1.1 (0.98)	0.2 (0.69)
10. Waves ‘hello’ and ‘goodbye’	Q	0.7 (0.89)	0.1 (0.24)
11. Uses common gestures	Q	0.4 (0.77)	0.2 (0.69)
12. Combines gestures with vocalizations to enhance communication	Q	1.0 (1.19)	0.2 (0.72)
13. Uses tone of voice appropriately to emphasize content of speech	Q	1.1 (1.00)	0.1 (0.24)
14. Comments on other people’s emotions	F	1.9 (0.75)	1.2 (0.78)
15. Looks when he/she is called or praised	F	0.9 (0.87)	0.3 (0.53)
16. Looks where another person is looking or pointing	F	1.2 (0.82)	0.2 (0.53)
17.** Shows pleasure in shared interactions**	F	1.1 (0.81)	0.1 (0.29)
18. **Uses facial expressions that are appropriate to the situation**	F	1.4 (0.85)	0.1 (0.38)
19. Resists affection from familiar people	F	0.8 (0.86)	0.3 (0.63)
20. Shows inappropriate affection towards unfamiliar people	F	0.5 (0.87)	0.0 (0.17)
21. **Has difficulty interacting with peers**	F	1.8 (0.93)	0.2 (0.39)
22. Says socially inappropriate things	F	1.5 (0.85)	0.3 (0.64)
23. Attends to parts of sentences and misinterprets whole	F	1.5 (0.86)	0.2 (0.61)
Restrictive Repetitive Behaviors			
24. Gets upset over small changes in routine	F	1.4 (1.00)	0.3 (0.47)
25. Has difficulty being flexible	F	1.7 (0.96)	0.4 (0.49)
26. Resists trying out new things	F	1.5 (0.99)	0.5 (0.51)
27.** Insists on doing things the same way each time**	F	1.6 (0.95)	0.1 (0.36)
28. **Is fixated on certain topics or activities and unable to move on**	F	1.8 (0.92)	0.1 (0.33)
29. Has an unusually narrow range of interests	F	1.8 (0.99)	0.0 (0.17)
30. Repeats/echoes what others say	F	0.8 (0.94)	0.1 (0.24)
31. Insists on saying words and phrases over and over	F	1.1 (1.12)	0.0 (0.17)
32. **Has mannerisms or odd ways of moving her/his hands or fingers**	F	1.1 (1.04)	0.1 (0.38)
33.** Makes repetitive movements**	F	1.1 (1.15)	0.0 (0.17)
34. **Attempts to harm him/herself**	F	0.3 (0.68)	0.0 (0.17)
35. Over-reacts to common smells	F	1.0 (0.96)	0.0 (0.24)
36. **Over-reacts to noise or sounds**	F	1.4 (0.96)	0.0 (0.17)
37.** Over-reacts to touch or being held**	F	0.9 (0.94)	0.1 (0.24)
38. Has sensitivities to certain food textures	F	1.6 (1.11)	0.2 (0.43)
Mood and Anxiety			
39. Cries over minor annoyances and hurts	F	0.9 (0.99)	0.2 (0.43)
40. Is irritable and whiny	F	1.2 (0.97)	0.4 (0.49)
41. **Worries about things**	F	1.5 (0.93)	0.7 (0.64)
42. **Is tense or anxious**	F	1.4 (0.94)	0.4 (0.55)
43. Clings to adults or is too dependent on them	F	0.8 (0.95)	0.1 (0.24)
44.** Is anxious in social situations**	F	1.4 (0.95)	0.2 (0.43)
45. Appears sad	F	0.8 (0.71)	0.4 (0.55)
46. **Is fearful of specific objects or situations**	F	0.9 (0.82)	0.0 (0.17)
47. Has sleep problems**	F	1.4 (1.11)	0.3 (0.62)
Self-regulation			
48. Has difficulties waiting his/her turn	F	1.3 (1.00)	0.2 (0.43)
49. **Acts without thinking**	F	1.6 (0.93)	0.4 (0.50)
50. Acts recklessly	F	0.8 (0.94)	0.1 (0.36)
51. **Switches quickly from one topic or activity to another**	F	1.3 (0.93)	0.3 (0.47)
52. Has difficulties playing or engaging in leisure activities quietly	F	1.0 (1.00)	0.1 (0.33)
53. Fidgets	F	1.5 (1.02)	0.4 (0.56)
54. Has difficulty remaining seated	F	1.2 (1.07)	0.2 (0.41)
55. **Is excessively active**	F	1.0 (1.10)	0.3 (0.53)
Challenging behavior			
56. Is verbally aggressive towards other children or adults	F	0.6 (0.83)	0.1 (0.24)
57. **Is physically aggressive towards other children or adults**	F	0.4 (0.60)	0.1 (0.29)
58.** Reacts with aggression when he/she is upset or stressed**	F	0.9 (0.98)	0.2 (0.50)
59. Throws things inappropriately	F	0.5 (0.7)	0.1 (0.29)
60. Runs away	F	0.3 (0.57)	0.0 (0.17)
61. Takes or grabs things that belong to others	F	0.6 (0.8)	0.1 (0.29)
62. **Has temper outbursts or tantrums**	F	1.0 (0.87)	0.1 (0.36)

Anchor response options: Frequency (F)—never, sometimes, often, very often; Quality (Q)—not at all, with support, with some reminders, without help

Bold indicates ABI-S items; *becomes 2 items in revised version; **moved from Self Regulation. Bold items appear in both the ABI and ABI-S. Bold items appear only in ABI.

## Methods

### Ethical Practices

Institutional Review Boards^1^ approved the study protocol and its amendments. Participants, their parents (for participants < 18 years old), or legally authorized representatives provided written informed consent before participating in the study. Minors who were participants provided assent. The study was registered at clinicaltrials.gov, NCT02299700.

### Study Samples

The study enrolled males and females aged ≥ 6 years with a confirmed diagnosis of ASD based on clinical examination including the Autism Diagnostic Observation Schedule, 2nd edition (ADOS-2) (Lord et al. 2012). These participants were requested to maintain ongoing behavioral and/or pharmacologic treatments during the course of the study, and it was expected that some changes would be seen in behaviors over the 8–10 week period as a result of these interventions or other prevailing events. Participants either lived with a parent or primary caregiver, spent at least 3 h a day for at least 4 days each week, or at least three weekends a month with a parent or primary caregiver. Other components of the broader study (Ness et al. 2019) required participation in a biosensor task battery resulting in exclusion criteria which included a measured composite score on the Kaufmann Brief Intelligence Test-2 (KBIT-2) (Kaufman and Kaufman 2004) of < 60 during screening (or other recent IQ evaluation), history of or current significant medical illness, and psychological and/or emotional problems that would render informed consent invalid or limit participant ability to comply with study requirements, based on clinical judgment.

The study also enrolled volunteer control participants through advertising across all sites. This sample included typically developing (TD) males and females aged ≥ 6 years with a score in the normal range on the SCQ, no DSM-5 defined major mental health disorder, no significant medical illness as assessed by the MINI-Kid v7.0.0 (Sheehan et al. 2010), and not taking psychotropic medication. This TD cohort provided normative data for comparison with ASD participants.

**Fig. 3:**
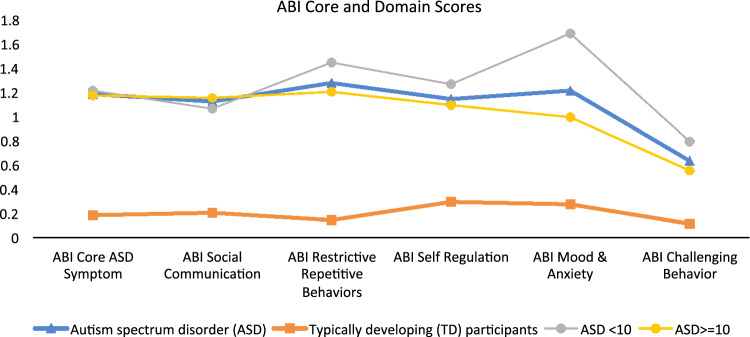
Mean ABI Scale Scores for ASD and TD participants at baseline based on Caregiver Responses to ABI

The study population comprised 144 participants with ASD and 41 TD participants. The majority were male (ASD 77.8%; TD 65.9%), consistent with higher male:female ratio in ASD (Loomes et al. 2017); their mean age was 15 years (Table 2). Mean (SD) ADOS Calibrated Severity Score (CSS) for the ASD participants was 7.6 (1.7), IQ was 99.2 (19.6), and all were verbal, based on parents report of language ability. Mean ABI scale scores at baseline showed clear differences between the ASD and TD cohorts for all domains, consistent with expectations (Table 1), and between younger (≤ 10 years old) and older (> 11) ASD participants for the Self-Regulation domain (Fig. 3).

**Table 2:** Participant characteristics

Characteristic	ASD Total N = 144	TD N = 41	*p*
Gender, n (%)			
Male	112 (77.8)	27 (65.9)	0.12
Female	32 (22.2)	14 (34.1)	
Age (years)			
Mean (SD)	14.6 (7.8)	16.3 (13.18)	0.30
Median (range)	12.5 (6–54)	11.0 (6–63)	
Age distribution, years, n (%)			
6–11	58 (40.3)	21 (51.2)	
12–17	46 (31.9)	13 (31.7)	
18–30	34 (23.6)	1 (2.4)	
≥ 31	6 (4.2)	6 (14.6)	
Race, n (%)			
White	118 (81.9)	34 (82.9)	0.88
Black or African American	6 (4.2)	2 (4.9)	
Asian	4 (2.8)	0	
Multiple	10 (6.9)	3 (7.3)	
Other	4 (2.8)	0	
Missing/unknown	2 (1.4)	2 (4.9)	
ADOS CSS Total Score, mean (SD)	7.6 (1.7)	–	–
KBIT-2 IQ Composite Score, mean (SD)	99.2 (19.6)	–	–

*ADOS CSS* Autism Diagnostic Observation Schedule, 2nd edition, comparison score; *KBIT-2* Kaufmann Brief Intelligence Test-2

P-values for gender and race (white vs. non-white) based on Chi square test

P-value for age based on two-sample t-test

### Study Design

This was a non-intervention, multicenter study, conducted from 06 July 2015 to 14 October 2015 at 9 study sites in the US.

The study consisted of a 14-day screening phase followed by an 8-to-10-week data-collection phase during which parents/caregivers of ASD participants completed the ABI/ABI-S at baseline, 3–5 days later, at week 4, and study endpoint (8–10 weeks). Parents of TD participants completed the ABI at a single visit. Parents/caregivers of ASD participants completed the remaining instruments at baseline, midpoint, and study endpoint.

### Instruments

#### Autism Behavior Inventory (ABI)

The ABI v 1.0 presented in the study consisted of 73 items across 5 domains as follows: (a) Social Communication (b) Restrictive Behaviors and co-occurring symptom domains of (c) Mood and Anxiety (items related to sadness, irritability, worry, and anxiety), (d) Self Regulation (inattentiveness, impulsiveness, overactivity, and sleep issues); and Challenging Behavior (verbal and physical aggression, tantrums, absconding). Caregivers were asked to respond to items on two of 4 possible dimensions: Quality (how *well* behaviors are carried out), Context (the *variety* of situations in which the behaviors occur), Frequency or Intensity (not present to very severe). “Quality and Context” and “Frequency and Intensity” are usually paired together. The data obtained in this study was used to evaluate the utility of 2 response dimensions, and to evaluate the performance of items selected for the ABI v 1.1. The ABI (v1.1) contains 61/73 items, plus 1 new item (Table 4). The analysis represented here reflects the ABI (v 1.1) 61 items.

#### Autism Behavior Inventory—Short Form (ABI-S)

The ABI-S contains a subset of 24 items across each of the five domains in the ABI.

#### Aberrant Behavior Checklist (ABC)

ABC (Aman et al. 2004; Aman and Singh 2017) is a 58-item behavior rating scale used to measure behavior problems across five subscales: Irritability, Social Withdrawal, Stereotypic Behavior, Hyperactivity/Noncompliance, and Inappropriate Speech. Items are rated on a 4-point Likert scale (ranging from 0 [not at all a problem] to 3 [The problem is severe in degree]), with higher scores indicating more severe problems. The ABC has recently been validated for use in ASD (Kaat et al. 2014).

#### Zarit Burden Interview (ZBI)

ZBI—short version (Zarit et al. 1980) is a scale with 22 items designed to assess psychological burden experienced by caregivers. Items ask how the caregivers feel, and responses range from 0 to 4 (never to nearly always). The ZBI has been used to assess burden among caregivers of individuals with ASD (Cadman et al. 2012; Hérbert et al. 2000).

#### Social Responsiveness Scale (SRS-2)

Social Responsiveness Scale 2™ (SRS-2) (Constantino et al. 2003) identifies presence and severity of social impairment due to ASD. It contains 65 items intended to assess social communication and restricted and repetitive behaviors. Three forms are available, dependent on the age of the individual with ASD.

#### Child and Adolescent Symptom Inventory—CASI-Anxiety

CASI-Anxiety (Hallett et al. 2013; Sukhodolsky et al. 2008) is a 21-item anxiety scale that has been recommended as a possible outcome measure for anxiety symptoms in ASD (Lecavalier et al. 2014).

#### Repetitive Behavior Scale—Revised RBS-R Parent

RBS-R (parent) (Bodfish et al. 1999) is a 43-item report scale to indicate occurrence of repetitive behaviors and degree to which a behavior is a problem on a range from 0 to 3 (does not occur to severe problem).

### Psychometric Analyses

Descriptive statistics were used to assess the measurement properties of the ABI, including evaluation of response variability and floor and ceiling effects. Comparison of the ABI responses using a single vs. dual response option was made using Pearson’s correlation coefficient of items in a domain scored on combined response option compared with first response option. Each domain was assessed by Cronbach’s alpha and item-total correlations for internal consistency. A domain was generally considered to have adequate internal consistency if Cronbach’s alpha was > 0.70.

Test–retest reliability at baseline and 3-to-5 days later was evaluated using Intraclass Correlation Coefficient (ICC). An ICC value of 0.70 or greater was considered evidence of acceptable test–retest reliability for subscale means and for use in detecting group mean differences. This time period was selected as a compromise. A shorter time period between test and retest may increase the likelihood of memory effects. This shorter period was selected, since the recall period for the ABI is one week, and therefore caregivers would be reporting on some of the behaviors within the same time frame as the original completion.

Scale-level convergent and discriminant validity were assessed by examining Pearson correlation coefficients between ABI domain scores and scores from related instruments at baseline. Convergent validity was established if at least moderate correlation (> 0.40) was observed between established measures and ABI scales hypothesized to measure the same or similar construct, and discriminant validity if correlations were lower than 0.40.

### Exploratory Analysis of Change Over Time

Though this was a non-interventional study, participants were instructed to continue treatment as usual, and so change in reported behaviors was measured at baseline and endpoint (8 weeks). Sensitivity to change was explored by comparing parent-reported change scores of participants whose health state did not change during this time to those who showed improvement. Two definitions of improvement in health state were evaluated and included improvement in at least one category on the: (1) SRS-2 Total Score severity category (within normal limits, mild, moderate, and severe) and (2) ZBI item 22 on overall caregiver burden (not at all, a little, moderately, quite a bit, and extremely). These measures were selected for comparison based on observed correlations between domains of interest. The magnitude of each within-group change was assessed using a paired *t*-test. Within-group effect sizes (ESs) were computed as the ratio of the mean change score to the pooled standard deviation of the change scores.

## Results

### Response Options

Pearson’s correlation coefficient between single or dual item responses was high (0.95–0.99) for each of the domains of the ABI. Since this suggested limited utility of the dual response, further analysis took place based on scores generated from a single response option.

### Internal Consistency

Internal consistency was high across domains, with Cronbach’s alpha ranging from 0.84 to 0.89 in the ABI (ABI-S: 0.69–0.79). Three items were identified through item-total correlations as having low correlation with their hypothesized domain score (r < 0.4 after adjusting for overlap), and when deleted resulted in a higher coefficient alpha for the remaining items in their hypothesized domain. Two of these items—*Shows inappropriate affection to unfamiliar people* [ABI 24] and *Attempts to harm him*/*herself* [ABI 39]—were identified previously as low prevalence behaviors but were maintained after review by clinical experts due to their seriousness when present. Wording changes were made to both of these items in order to provide clarification for future versions, as Cognitive Interviews also revealed potential confusion (Pandina et al. 2018). The correlation between *Has sleep problems* and the Self-Regulation domain adjusted for overlap was (0.38). This item was moved to the Mood and Anxiety domain.

### Test–Retest Reliability

Test–retest reliability of each domain score on the ABI 3–5 days after baseline was excellent, with ICC values ranging from 0.84 to 0.93. ABI-S test–retest reliability was good (0.77–0.88). Means did not change significantly between test and retest (Table 3).

**Table 3:** Test–retest correlations for all ABI/ABI-S Subscales based on Caregiver Responses to ABI

	Core ASD n = 88	Social Communication n = 88	Restrictive Repetitive Behaviors n = 88	Mood & Anxiety n = 88	Self-regulation n = 88	Challenging Behavior n = 88
ICC estimate	0.89	0.88	0.86	0.84	0.89	0.93
ABI						
*p*-value	< 0.0001	< 0.0001	< 0.0001	< 0.0001	< 0.0001	< 0.0001
95% CI	(0.84, 0.93)	(0.82, 0.92)	(0.79, 0.91)	(0.76, 0.89)	(0.83, 0.92)	(0.90, 0.95)
ICC estimate	0.85	0.77	0.82	0.84	0.88	0.88
ABI-S						
*p*-value	< 0.0001	< 0.0001	< 0.0001	< 0.0001	< 0.0001	< 0.0001
95% CI	(0.78, 0.90)	(0.67,0.85)	(0.74, 0.88)	(0.77, 0.89)	(0.82, 0.92)	(0.83 0.92)

*p*-value for difference from a one-sample *t*-test. Pearson correlation based on test and retest values. ICC was 2, 1 variant

### Convergent and Discriminant Validity

Pearson’s correlations between ABI domains and comparison instruments were strongly positive (Table 4), demonstrating good convergent validity between subscales. The numbers in Table 4 that appear in bold font demonstrate examples of pre-specified variables showing convergent validity for subscales assessing analogous constructs. Correlations between ABI domains and ADOS were small (Table 4).

**Table 4:** Pearson correlations between ABI domains and related instruments (N = 139 ASD participants)

Instrument	ABI domain					
	Core ASD Symptoms	Social Communication	Restrictive Repetitive Behaviors	Mood & Anxiety	Self-regulation	Challenging Behavior
ADOS-2 Comparison Score	0.17	0.20	0.09	− 0.14	0.04	− 0.02
SRS-2 (Parent)						
Total Score	**0.81**	**0.66**	0.73	0.49	0.43	0.31
Social communication and interaction	**0.80**	**0.69**	0.68	0.46	0.39	0.27
Restricted interests and repetitive	0.72	0.49	**0.75**	0.50	0.49	0.38
CASI-4R Anxiety Scale Score	0.53	0.32	0.59	**0.77**	0.33	0.21
RBS-R Overall Score	0.67	0.40	**0.76**	0.45	0.51	0.40
ABC—Community						
Irritability	0.49	0.28	0.56	0.57	0.64	**0.76**
Social withdrawal	**0.66**	0.66	0.47	0.33	0.12	0.14
Stereotypic Behavior	0.57	0.39	**0.59**	0.34	0.53	0.41
Hyperactivity/noncompliance	0.43	0.24	0.51	0.35	**0.88**	0.55
Inappropriate speech	0.56	0.33	**0.65**	0.39	0.64	0.48
ZBI Total Score	0.29	0.14	0.36	0.41	0.35	0.47
ABI-S	0.92	0.84	0.93	0.91	0.93	0.94

Bold indicates pre-specified variables showing convergent validity

*ABC-Community* Aberrant Behavior Checklist-Community; *ADOS-2* Autism Diagnostic Observation Schedule, 2nd edition, comparison score; *CASI-4R Anxiety* Child and Adolescent Symptom Inventory 4R, Anxiety Subscale, *RBS-R* Repetitive Behavior: Repetitive Behavior Scale—Revised; *SRS-2* Social Responsiveness Scale-2; *ZBI* Zarit Burden Interview SRS-2, *ABI-S* Autism Behavior Inventory—Short form

Discriminant validity was generally established in that the correlations between analogous constructs exceeded correlations between non-analogous constructs. For example, the correlation between the CASI-Anxiety score and the Mood & Anxiety Domain (*r* = 0.77) exceeded correlations between the CASI-Anxiety score and the remaining ABI domains. An exception was that the SRS-2 Social Communication and Interaction domain was unexpectedly highly correlated with the Restrictive Repetitive Behaviors domain from the ABI (*r* = 0.68).

The ZBI Total Score was moderately correlated to all ABI domains except Social Communication. Pearson’s correlation coefficient for the ABI with the ABI-S domains are shown in the final row of Table 4. The relationship between the ABI-S and the other parent rated scales was similar to the ABI (e.g. Core Symptoms ABI-S with SRS total 0.80, Mood & Anxiety ABI-S with CASI-Anx 0.76, Self regulation ABI-S with ABC Hyperactivity and Non-Compliance 0.83).

### Change Over the Duration of the Study

Supplemental Table 1 presents changes in ABI and other scales observed over the course of the study. A trend towards improvement was seen across all scales over the 8–10 week period.

### Change Over Time

Changes in ABI scores between baseline and study endpoint were compared with changes in SRS Total Score severity category and changes in overall parent burden (ZBI item 22) in an exploratory analysis of change over time. Subscales responsive to improvement should have a large positive effect size for participants experiencing improvement and a smaller (close to 0) effect for those who did not experience change.

Participants showing improvements in ASD severity based on category change in SRS-2 Total Scores showed analogous ABI domain score improvements in Core ASD Symptoms, Social Communication, and Restrictive Repetitive Behaviors (moderate to large within-group effect sizes of 0.63, 0.50, and 0.41, respectively) (Table 5). And, participants showing improvements in overall burden based on category change in ZBI showed analogous ABI domain scores improvements in Restrictive Repetitive Behaviors, Mood and Anxiety, Self Regulation, and Challenging Behavior (mild-to-moderate within-group effect sizes of 0.39, 0.27, 0.29, and 0.27 respectively). In both cases, these effects were not observed in groups with no documented change or who had worsened.

**Table 5:** Summary of effect sizes of selected Patient Reported Outcomes at endpoint visit

	SRS-2 Total Score Severity			ZBI 22 Overall Burden		
	Improved (n = 28)	No change (n = 85)	Worsened (n = 13)	Improved (n = 27)	No change (n = 84)	Worsened (n = 16)
ABI						
Core ASD Symptoms	*0.63*	*0.28*	*− 0.34*	0.33	0.29	0.02
Social Communication	*0.50*	*0.20*	*0.06*	0.11	0.32	0.01
Restrictive behavior	*0.41*	*0.20*	*− 0.61*	*0.39*	*0.11*	*0.02*
Mood and Anxiety	0.39	0.13	**−** 0.19	*0.27*	*0.13*	*0.04*
Self Regulation	0.13	0.33	**−** 0.54	*0.29*	*0.14*	*0.25*
Challenging Behavior	0.19	0.13	**−** 0.39	*0.27*	*0.01*	*0.08*
ABI-S						
Core ASD Symptoms	*0.51*	*0.33*	*− 0.19*	0.51	0.25	0.28
Social Communication	*0.45*	*0.19*	*0.12*	0.17	0.24	0.35
Restrictive Behavior	*0.32*	*0.29*	*− 0.38*	*0.58*	*0.14*	*0.07*
Mood & Anxiety	0.26	0.07	**−** 0.43	*0.18*	*0.03*	*− 0.03*
Self Regulation	0.10	0.31	**−** 0.59	*0.21*	*0.13*	*0.26*
Challenging Behavior	0.13	0.18	**−** 0.52	*0.30*	*0.01*	*0.00*
SRS-2						
Total Score				0.20	0.25	0.55
Social Comm. Interaction				0.21	0.24	0.65
Rest. Int. Rep. Behaviors				*0.15*	*0.19*	*0.29*

By convention, effect sizes for change groups whose mean change from baseline are in the direction of worsening are denoted with a negative sign. Effect sizes for no change are taken from the pairwise analysis of improved versus no change and differ slightly from effect sizes from the pairwise analysis of worsened versus no change due to differences in the estimation of the pooled SD. Highlighted (italic font) values indicate domains that correlated at baseline and for which change over time was expected to correlate

## Discussion

Internal consistency (α) was high for all ABI domains, and test–retest reliability was excellent based on established benchmarks (good = 0.64–0.74, excellent ≥ 0.75) (Cicchetti and Sparrow 1981). Strong positive correlations were observed with analogous parent-reported subscales, and only mostly moderate correlations with subscales assessing divergent constructs. Thus, ABI and the ABI-S allow for the potential to complete one instrument in place of discrete alternatives commonly used in treatment outcome studies and clinical drug trials.

Analysis of response option performance indicated that scores obtained based on combination of 2 response options, such as frequency and intensity, were very closely related and it appears, with this observation, that the second response may be redundant. We introduced the dual response options with the intention that this would result in increased sensitivity to change. While this is still possible, we cannot draw this conclusion based on the available observations. Given the increased burden to caregivers, essentially doubling the items on the scale for completion, and the potential for increased complexity, we opted to finally select a single response anchor: Quality or Frequency. Use of a single anchor response and possible response options were tested and received a favorable response from parents and caregivers in the cognitive interview study (Pandina et al. 2018).

The ABI-S also shows good psychometric properties. The intention is to use the short form of the ABI more frequently over the course of a clinical study to further reduce caregiver burden. Further data on change over time in response to intervention on the ABI compared to the ABI-S is required to determine which version is most useful as an outcome measure.

Our preliminary change over time analyses suggest that the ABI changes were consistent with corresponding changes across multiple categories in other parent-reported scales that occur over an 8–10 week period. This empirical, anchor-based approach is consistent with some FDA guidance for patient-reported outcome measures (FDA 2009). Based on observed correlations, the SRS-2 was selected as an appropriate anchor for the Social Communication and Restrictive Repetitive Behaviors domains, while parent burden assessed on the ZBI (Item 22 Overall Burden) was an appropriate anchor for the Restrictive Repetitive Behaviors, Mental Health, Self-regulation, and Challenging Behavior domains since it was correlated with these scales at baseline.

Scores on the ABI were associated with changes of at least one severity category in SRS-2. Effect sizes for the group who improved exceeded 0.40 for both Social Communication and Restrictive Repetitive Behaviors domains, whereas the largest effect size of participants whose SRS-2 severity did not change was 0.29. Reductions in the ABI were also associated with reductions of at least one category in parent burden, indicating that as symptoms were reducing, parent burden was reported as lower. This was an exploratory approach which aimed to link parent-reported change in child behavior to a meaningful quality-of-life indicator (in this case, level of burden felt by parents in caring for their children with ASD). In this group, burden was not related to Social Communication skills, but did relate to behaviors reported in other domains. However, we note that this approach is limited by the issue of “source or method variance” (Campbell and Fiske 1959; Podsakoff et al. 2003) (i.e. insofar as change is concerned, we cannot determine with certainty whether the parents were accurately reporting genuine alterations in behavior or *perceived* changes). We acknowledge the limitation, and we are currently evaluating the ABI’s performance in a placebo-controlled, randomized clinical trial of a rational therapeutic agent. This trial also includes clinician-reported measures, such as the Clinical Global Impressions Scale (CGI) (Arnold et al. 2000). In the meantime, these analyses suggest that ABI is sensitive over time in a manner that is congruent with other clinical measures.

This study examined a well-characterized sample of participants with a clinical diagnosis of ASD confirmed by ADOS. However, there was a poor correlation between ABI, which is intended to measure changes in “states over time” based on parent observation in natural settings, and ADOS, a tool principally designed to capture patient “traits” and evaluate the presence/absence of ASD based on direct assessment (usually lasting an hour or less) in clinical settings. Discrepancies between parent report and direct assessment have been observed in other studies (see review by Achenbach et al. 1987; Kaat et al. 2014; Mirenda et al. 2010; Sturm et al. 2017), and the ADOS specifically (Mazurek et al. 2018). This further suggests that behaviors specific to autism and critical for *diagnosis* may not be the same as those that indicate changes in symptom severity over time. For example, the items in the ABI social communication domain may be more commensurate with measures of adaptive behavior.

The ABI was not developed as a diagnostic tool. It was designed to focus on behaviors that might be targets for change in ASD rather than those that might demonstrate greatest sensitivity and specificity for diagnosis. Therefore, we did not include comparison participants with intellectual disability or communication disorders, which were often a typical part of the validation process in the past for diagnostic scales. However, the ABI did show good discrimination between ASD and TD groups, suggesting that it can be used to define ASD symptom severity for use as an inclusion criterion in clinical trials.

Taken together, our findings support use of the ABI as a clinical endpoint with the potential to identify and measure short term change in parent-reported behaviors. Our methodological approach included statistical and clinical review of items and careful selection and consideration of response scales provide appropriate response options for parents (Fok and Henry 2015). The 1-week time period for reporting, compared to other scales with longer recall, may enhance suitability of the scale for this purpose.

The cohort in this validation study covered a broad range of participant ages and ASD severity levels. However, it is likely that, given other requirements of the study, this group of individuals had less extreme challenging behaviors, which would explain near floor effects in reported items such as elopement and physical aggression. The lack of representativeness of this group is the reason for retaining these items. Cognitive interviewing indicated the appropriateness of these items. Further psychometric validation in populations including more minimally verbal participants and those with a broader range of challenging behaviors is planned. In addition, our sample included only individuals over the age of 6 years, whereas the ABI items were designed to be suitable for children aged 3 years and above. There were also fewer individuals over 18, and the cohort was of average IQ, and predominantly Caucasian. Further studies with younger children and older adults, as well as a sample with greater diversity in race/ethnicity and IQ are also planned. Translation and validation of the ABI in other languages and cultures are also in progress.

Though the ABI has been used by different groups of raters, there are currently insufficient interrater reliability data between caregivers for statistical analysis. A clinician-rated version is in development and will be reported elsewhere. A self-report version for individuals capable of responding is also planned. The ability of individuals with ASD to self-report and how this differs from a parent perspective are both important to determine in future research.

The ABI and ABI-S are available without charge for academic, research, and professional use, subject to terms and conditions. They can be downloaded in the USA from https://www.janssenmd.com/ (in the tools/psychiatry section) and accessed outside the USA via email request to autismbehaviorinventory@its.jnj.com.

Limitations of the study include a modest-size sample (for psychometric purposes), reliance on existing interventions to monitor change, and the source or method-variance issue.

In summary, the ABI continues to demonstrate good psychometric properties—sound structure and good reliability and validity—in two clinical populations of individuals with ASD. There is some evidence of change in the short term, congruent with changes in other measures, which is critical for clinical endpoint assessments. The next line of investigation is the use of ABI as a parent-reported measure in ASD treatment studies.

## Electronic supplementary material

Below is the link to the electronic supplementary material.

Supplementary material 1 (DOCX 29 KB)

## Footnotes

1 Investigator name: IRB name, IRB Reference Number: Yvette Janvier, MD and Christopher Smith, PhD: Sterling Institutional Review Board, 5004C-001 and 5004C-002; Russell Tobe, MD: Nathan Kline Institutional Review Board, 1517-00; Geraldine Dawson, PhD: Duke University Institutional Review Board, Pro00064177; Judith S. Miller, PhD: Children’s Hospital of Philadelphia Institutional Review Board, 15-011867; Bryan King, MD: WIRB Institutional Review Board, 1158399; Frederick Shic, PhD: Yale University Institutional Review Board, 1510016645; Jean Frazier, MD: University of Massachusetts Institutional Review Board, H000088176; Bennett Leventhal, MD: University of California San Francisco Institutional Review Board, 144522.
