# Supplementary material for: Clinical Validation of the Autism Behavior Inventory: Caregiver-Rated Assessment of Core and Associated Symptoms of Autism Spectrum Disorder
Source: J Autism Dev Disord. 2019 Mar 19;50(6):2090–101. doi: 10.1007/s10803-019-03965-7 (PMC7261279; doi:10.1007/s10803-019-03965-7)
Supplement: Supplementary file 1 — Supplementary material 1 (DOCX 29 KB) [file 10803_2019_3965_MOESM1_ESM.docx]

**Table 1. Change from Baseline to Study Endpoint in ABI and Other Scales**

Change from Baseline

Scale or Subscale Base ---------------------------------------------

Analysis Time Point N Mean SD Med Min Max Mean N Mean SD Med Min Max

--------------------------------------------------------------------------------------------------------------------------------------

ABI Social Communication

Baseline 140 1.12 0.450 1.09 0.0 2.4

Endpoint 129 1.05 0.490 1.00 0.1 2.4 1.12 127 -0.07 0.278 -0.05 -1.0 0.7

ABI Restrictive Repetitive

Baseline 140 1.27 0.610 1.20 0.1 2.8

Endpoint 129 1.22 0.590 1.13 0.0 2.9 1.29 127 -0.06 0.359 0.00 -1.4 0.7

ABI Mood & Anxiety

Baseline 140 1.14 0.601 1.00 0.0 2.8

Endpoint 129 1.09 0.603 1.00 0.1 2.8 1.15 127 -0.06 0.398 0.00 -1.2 1.7

ABI Self-Regulation

Baseline 140 1.21 0.760 1.13 0.0 3.0

Endpoint 129 1.16 0.739 1.00 0.0 3.0 1.23 127 -0.08 0.424 0.00 -1.8 1.1

ABI Challenging Behavior

Baseline 140 0.63 0.557 0.57 0.0 2.6

Endpoint 129 0.61 0.610 0.57 0.0 2.7 0.64 127 -0.02 0.302 0.00 -0.9 1.1

SRS Total Score

Baseline 142 74.6 10.94 74.0 48 105

Endpoint 136 73.1 10.67 72.0 47 103 74.6 135 -1.5 5.41 -1.0 -17 10

ABC Subscale

Irritability/Agitation

Baseline 143 9.6 8.70 6.0 0 38

Endpoint 136 9.3 8.55 7.0 0 36 9.8 136 -0.5 4.77 0.0 -23 14

ABC Lethargy/Social Withdrawal

Baseline 143 11.2 8.54 10.0 0 39

Endpoint 136 10.1 8.11 8.0 0 36 11.2 136 -1.1 4.37 -1.0 -15 14

ABC Stereotypic Behavior

Baseline 143 5.1 5.13 4.0 0 21

Endpoint 136 5.3 5.11 4.0 0 19 5.2 136 0.0 3.30 0.0 -16 11

ABC Hyperactivity/Noncompliance

Baseline 143 15.7 11.59 13.0 0 43

Endpoint 136 14.0 10.42 12.0 0 45 15.8 136 -1.8 5.55 -1.0 -23 13

ABC Inappropriate Speech

Baseline 143 3.7 3.28 3.0 0 12

Endpoint 136 3.8 3.09 4.0 0 12 3.8 136 0.1 2.16 0.0 -6 5

CASI-4R Anxiety

Baseline 143 16.7 10.90 15.0 0 54

Endpoint 136 16.0 10.09 14.0 1 50 16.8 136 -0.8 6.48 -1.0 -25 22

RBS-R Overall Score

Baseline 143 27.9 21.52 21.0 1 99

Endpoint 136 26.8 20.61 21.0 1 111 28.1 136 -1.3 11.41 -1.0 -38 28

**Table 2. Participant Characteristics by Site**

|  | **All Sites** | **Site 1** | **Site 2** | **Site 3** | **Site 4** | **Site 5** | **Site 6** | **Site 7** | **Site 8** | **Site 9** |
| --- | --- | --- | --- | --- | --- | --- | --- | --- | --- | --- |
| N | 144 | 3 | 15 | 5 | 21 | 20 | 17 | 10 | 29 | 24 |
| Gender, n (%) |  |  |  |  |  |  |  |  |  |  |
| Male | 112 (77.8) | 3 (100.0) | 13 (86.7) | 2 (40.0) | 13 (61.9) | 17 (85.0) | 15 (88.2) | 10 (100.0) | 22 (75.9) | 17 (70.8) |
| Female | 32 (22.2) | 0 | 2 (13.3) | 3 (60.0) | 8 (38.1) | 3 (15.0) | 2 (11.8) | 0 | 7 (24.1) | 7 (29.2) |
| Age, years |  |  |  |  |  |  |  |  |  |  |
| Mean (SD) | 14.58 (7.830) | 13.67 (3.512) | 12.67 (5.512) | 13.00 (3.464) | 13.00 (6.834) | 15.40 (7.810) | 12.59 (4.048) | 10.80 (2.394) | 16.55 (10.186) | 17.50 (9.987) |
| Median | 12.5 | 14 | 11 | 14 | 12 | 14.5 | 12 | 11 | 16 | 17 |
| Range | (6.0; 54.0) | (10.0; 17.0) | (7.0; 28.0) | (8.0; 16.0) | (6.0; 38.0) | (6.0; 33.0) | (6.0; 19.0) | (7.0; 14.0) | (6.0; 54.0) | (6.0; 51.0) |
| Age, distribution years, n (%) |  |  |  |  |  |  |  |  |  |  |
| 6-11 | 58 (40.3) | 1 (33.3) | 8 (53.3) | 2 (40.0) | 10 (47.6) | 8 (40.0) | 5 (29.4) | 6 (60.0) | 10 (34.5) | 8 (33.3) |
| 12-17 | 46 (31.9) | 2 (66.7) | 5 (33.3) | 3 (60.0) | 7 (33.3% | 4 (20.0) | 9 (52.9) | 4 (40.0) | 7 (24.1) | 5 (20.8) |
| 18-30 | 34 (23.6) | 0 | 2 (13.3) | 0 | 3 (14.3) | 7 (35.0) | 3 (17.6) | 0 | 9 (31.0) | 10 (41.7) |
| 31+ | 6 (4.2) | 0 | 0 | 0 | 1 (4.8) | 1 (5.0) | 0 | 0 | 3 (10.3) | 1 (4.2) |
| Race, n (%) |  |  |  |  |  |  |  |  |  |  |
| White | 118 (81.9) | 1 (33.3) | 13 (86.7) | 3 (60.0) | 19 (90.5) | 18 (90.0) | 13 (76.5) | 7 (70.0) | 23 (79.3) | 21 (87.5) |
| Black or African American | 6 (4.2) | 0 | 0 | 0 | 0 | 0 | 1 (5.9) | 0 | 4 (13.8) | 1 (4.2) |
| Asian | 4 (2.8) | 0 | 1 (6.7) | 0 | 2 (9.5%) | 1 (5.0) | 0 | 0 | 0 | 0 |
| American Indian or Alaska Native | 1 (0.7) | 0 | 0 | 0 | 0 | 0 | 0 | 1 (10.0) | 0 | 0 |
| Multiple | 10 (6.9) | 1 (33.3) | 0 | 1 (20.0) | 0 | 1 (5.0) | 2 (11.8) | 1 (10.0) | 2 (6.9) | 2 (8.3) |
| Other | 3 (2.1) | 1 (33.3) | 1 (6.7) | 1 (20.0) | 0 | 0 | 0 | 0 | 0 | 0 |
| Not Reported | 2 (1.4) | 0 | 0 | 0 | 0 | 0 | 1 (5.9) | 1 (10.0) | 0 | 0 |
| Ethnicity |  |  |  |  |  |  |  |  |  |  |
| Hispanic or Latino | 19 (13.2%) | 0 | 0 | 2 (40.0) | 1 (4.8) | 3 (15.0) | 0 | 1 (10.0) | 6 (20.7) | 6 (25.0) |
| Not Hispanic or Latino | 123 (85.4) | 3 (100.0) | 15 (100.0) | 3 (60.0) | 20 (95.2) | 17 (85.0) | 16 (94.1) | 8 (80.0) | 23 (79.3) | 18 (75.0) |
| Not Reported | 2 (1.4) | 0 | 0 | 0 | 0 | 0 | 1 (5.9) | 1 (10.0) | 0 | 0 |
